# Dexamethasone release from hyaluronic acid microparticle and proanthocyanidin-gelatin hydrogel in sciatic tissue regeneration

**DOI:** 10.1007/s10856-023-06768-6

**Published:** 2024-01-11

**Authors:** Kazem Javanmardi, Hamideh Shahbazi, Ava Soltani Hekmat, Mehdi Khanmohammadi, Arash Goodarzi

**Affiliations:** 1 Department of Physiology, Fasa University of Medical Sciences, Fasa, Iran; 2 Skull-Based Research Center, Five Senses Health Research Institute, School of Medicine, Iran University of Medical Sciences, Tehran, Iran; 3 Faculty of Materials Science and Engineering, Warsaw University of Technology, Warsaw, Poland; 4 Department of Tissue Engineering, School of Medicine, Fasa University of Medical Sciences, Fasa, Iran

## Abstract

Biodegradable microparticles are useful vehicles for the controlled release of bioactive molecules in drug delivery, tissue engineering and biopharmaceutical applications. We developed dexamethasone (Dex) encapsulation into tyramine-substituted hyaluronic acid microparticles (Dex-HA-Tyr Mp) mediated by horseradish peroxidase (HRP) crosslinking using a microfluidic device and infollowing crosslinked gelatin (Gela) with proanthocyanidin (PA) as a semi-confined bed hydrogel for the repair of sciatic tissue injury. It was found that the simultaneous use of Dex-HA-Tyr Mp and cross-linked Gela-PA hydrogel improved the physical properties of the hydrogel, including mechanical strength and degradability. The designed composite also provided a sustained release system for Dex delivery to the surrounding sites, demonstrating the applicability of the fabricated hydrogel composite for sciatic nerve tissue engineering and regeneration. The encapsulated cells were viable and showed adequate growth ability and morphogenesis during prolonged incubation in Gela-PA/HA-Tyr Mp hydrogel compared to control conditions. Interestingly, histological analysis revealed a significant increase in the number of axons in the injured sciatic nerve following treatment with Dex-HA-Tyr Mp and injectable Gela-PA hydrogel compared to other control groups. In conclusion, the results demonstrated that fabricated Dex-loaded MPs and injectable hydrogel from biomimetic components are suitable systems for sustained delivery of Dex with adequate biocompatibility and the approach may have potential therapeutic applications in peripheral nerve regeneration.

**Graphical Abstract:**

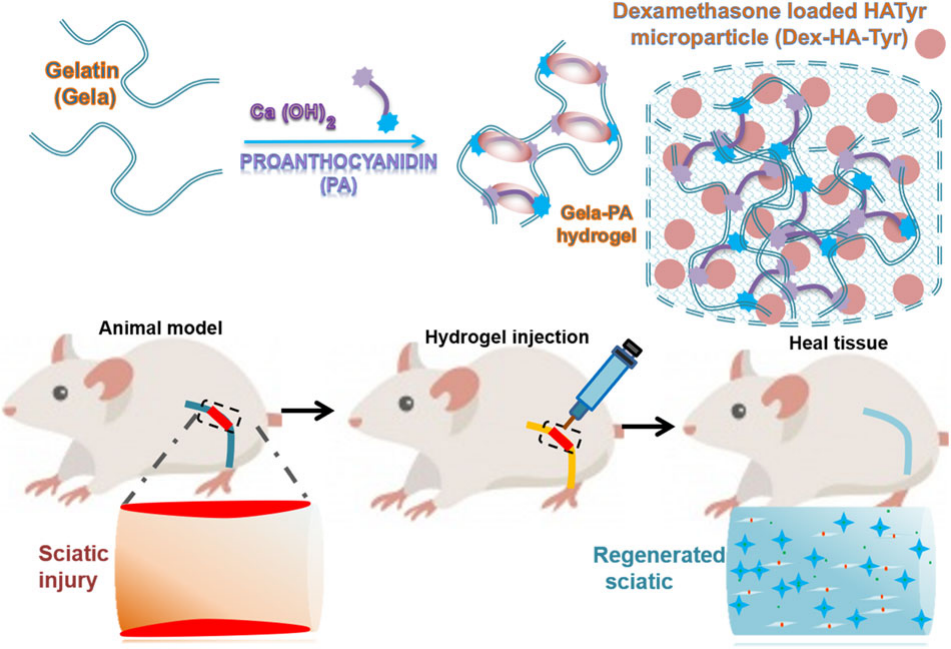

## Introduction

Peripheral nerve injury is one of the frequent types of neurological injuries, mostly caused by neuronal destruction, axonal network dysfunction, and vascular damage [1–3]. Additionally, functional loss of related body organs and the progression of related impairments occur as secondary damages due to the apoptosis and necrosis of nerve cells, release of toxins, and ischemia resulting from the inflammatory response [1, 3, 4]. Peripheral nerve repair is commonly managed through surgical procedures, combined with physical therapy, and the use of pharmaceuticals for pain management [1, 2, 5]. In recent years, significant progress in the fields of materials science and regenerative medicine has advanced alternative approaches to the regeneration and repair of damaged neural tissue [1, 2, 4]. This involves the utilization of engineered hydrogels that incorporate a combination of extracellular matrix-derived substrates and systems for controlled delivery of bioactive molecules [2–4, 6]. In this regard, injectable composite systems based on microparticles in hydrogels for controlled delivery of bioactive cargo act as a double protective barrier, guaranteeing a longer system lifespan and a more sustainable release with a significant delay in the initial burst release profile [4, 7–9]. Additionally, the injectable composites can be easily adjusted to irregularly shaped tissue due to their inherent flexibility and conforming properties [10–13]. Material selection should be made based on key technical features, such as degradation and diffusion characteristics, as well as a suitable structural form for cell adhesion and tissue integration [14–16]. Among these, naturally derived biopolymers, such as hyaluronic acid, gelatin, and fibrin, possess key technical features for drug delivery and can mimic the biological properties of tissue due to their specific integrin molecular recognition [14, 17–19].

Furthermore, several drugs and their associated biological carriers have been developed to facilitate nerve regeneration, featuring controlled drug release rates, and the majority of these treatments have demonstrated positive effects in animal models [20–22]. Among these, dexamethasone (Dex) stands out as a prominent anti-inflammatory glucocorticoid commonly used post-injury to mitigate neural inflammation and expedite the process of target organ reinnervation. In detail, the neurotrophic effects triggered by Dex at the site of the nerve injury result in a reduction of inflammatory cell infiltration and the production of inflammatory mediators. This reduction occurs through the inhibition of activated macrophages [20–23].

However, due to the adverse effects of systemic therapy such as cardiovascular disease, weight gain, and osteoporosis, its usage is partially restricted [20, 22, 23]. These limitations could be overcome by using injectable composite systems based on microparticles in hydrogels that enhance drug efficiency at the target site [21, 24–27]. We have applied Dex for a sustained delivery system to treat sciatic tissue injury by developing injectable hyaluronic acid (HA) derivative microparticles and a gelatin-based hydrogel scaffold to ensure the prolonged release of Dex at the injection site and prevent adverse effects of systemic therapy. Simultaneously, it promotes tissue regeneration with prepared extracellular matrix (ECM)-derived composite that regulates cellular behaviors through cellular-mediated degradation and subsequently tissue reorganization [4, 8, 14].

In our previous studies, HA-based microparticles were developed by making use of horseradish peroxidase (HRP)-mediated reaction in the presence of hydrogen peroxide (H_2_O_2_) as an electron donor which couple two nearby tyrosyl radicals via a carbon–carbon at the ortho positions or via a carbon-oxygen bond between the carbon atom at the ortho position and the phenoxy oxygen results in di-tyrosine crosslinks [26–29]. Here, we prepared HA-based microparticles loaded with Dex molecules. Subsequently, we prepared an injectable gelatin-based hydrogel scaffold containing microparticles through proanthocyanidin (PA)-mediated hydrogelation to minimize possible cytotoxic consequences or unexpected by-products that may arise from photoinitiator or organic solvent-mediated reactions [30–33]. The PA, a natural polyphenolic oligomeric flavonoid found in plants, has variety of inherent biological activities, including anti-inflammatory, antioxidant, antibacterial, and anti-calcification features [33, 34].

## Materials and methods

### Materials

Sodium HA, tyramine hydrochloride, water-soluble carbodiimide hydrochloride (WSCD), n-hydroxysuccinimide (NHS), horseradish peroxidase, 2-morpholinoethane sulfonic acid (MES) were purchased from Sigma Chemicals (St. Luis. MO, USA). The proanthocyanidin was obtained from Shari (Grape seed resource; Iran). The H_2_O_2_ aqueous solution, acetone, ethanol, and other chemicals were obtained from Dr. Mojallali Chemical Labs.

### Tyramine substituted hyaluronic acid

The HA-Tyr were synthesized according to the reported method by amide bond formation via carbodiimide-mediated condensation of the carboxyl groups of HA with the amino group of tyramine using WSCD and NHS in MES buffer [35–38]. The synthesized HA-Tyr was characterized by ^1^H-NMR spectroscopy in D_2_O at 30 °C, and the degree of substitution of the Ph residue in the substrates was determined by UV–Vis spectrophotometer (Shimadzu RF -5000) at 275 nm using a calibration curve established for known percentages of tyramine hydrochloride. The HA-Tyr was sterilized with 96% ethanol solution under a laminar flow hood and dissolved in phosphate buffer saline (PBS, pH = 7.4). The HRP and other chemicals were sterilized using 0.2 µm pore membrane filters to remove microbial contamination. The microfluidic device, connecting tubes, liquid paraffin, and lecithin were sterilized using a steam autoclave. In addition, adipose-derived stem cells (ADSCs) from rat tissue were cultured as model cells in Dulbecco’s modified Eagle’s medium (Gibco Technologies, Logan, UT) containing 10% (v/v) heat-inactivated fetal bovine serum, 100 µg/mL penicillin and streptomycin under physiological conditions.

### Fabrication of dexamethasone-loaded HA-Tyr microparticle

The HA-Tyr hydrogel microparticles were prepared in a coaxial double orifice spinneret designed in our laboratory [4, 28, 39]. Aqueous polymer solution was prepared from HA-Tyr 10 mg/mL, HRP 100 units/mL, and Dex 1 mg/mL and passed through the inner tube of the microfluidic nozzle using a syringe pump at 2.75 mL/h. Liquid paraffin containing lecithin 5% (w/v) was passed through the outer channel of the microfluidic nozzle using a second syringe pump at 120 mL/h. The HA-based droplets were crosslinked with the Tyr residues by diffusion of H_2_O_2_ into them and the HRP-mediated reaction proceeded spontaneously. After standing for at least 30 min, the microparticles were collected by centrifugation. The harvested microparticles were rinsed with excess PBS and passed through a 100 µm sieve to collect Dex-HA-Tyr Mps.

### Morphology and geometry of HA-Tyr microparticle

Average size of the prepared microparticles was quantified by geometric measurements using microphotographs taken with an inverted light microscope and processed using Image J software [4, 28, 39]. For this, the diameter of microparticles was measured for 100 randomly selected beads from triplicate independent experiments using the microscope images and Image J software (the U.S. National Institutes of Health). Finally, frequency distribution was calclulated to understand range of values occurs. To assess the morphology and microstructure of the hydrogel microparticles, the scanning electron microscopy (SEM) technique was used. For this, the microparticles were dried using an ethanol/water mixture at different concentrations (50%, 60%, 70%, 80%, 90%, 100%) for 10 min in each concentration. Then, the dried microparticles were coated with a 10 nm gold layer using K450X EMITECH coater (England) and the SEM images were taken using FE-SEM Mira 3 TESCAN, Czech Republic.

### Injectable gelatin-based hydrogel

Gela-based hydrogels were prepared by adding 0.5% (w/v) PA solution containing 0.05 M Ca(OH)_2_ which facilitates the penetration of PA and crosslinking of protein-based biopolymers. For this, the precursor gelatin hydrogel solution (24 mg/mL) was maintained at 37 °C for 1 h and then Dex-HA-Tyr Mp and PA solution were added to this polymer solution in a volume ratio of 10:1:1 (v/v) and further incubated for hydrogelation. The prepared hydrogels were used for biophysical and biochemical characterization in vitro and subsequently for in vivo studies.

### FTIR spectra measurements

Fourier transform infrared spectroscopy (FTIR) were done using a Bruker Equinox 55LS 101 series instrument (Germany) with the adjusted resolution of 4 cm^−1^ within the 400–4000 cm^−1^ range to assess the functional groups of the samples and the interactions in prepared hydrogels.

### Physical properties of hydrogels

The hydrogels with a diameter of ~12 mm and a height of ~8 mm were prepared in a cylindrical PDMS module and examined in triplicate. The compressive strength of the hydrogels was determined using GOTECH GT-TCS-2000 with a 100 kN load cell at constant deformation rate of 2 mm/min to assess the mechanical properties of the samples. The mechanical strength was calculated by the division of resistance force per surface area of the cylindrical-shaped hydrogel.

For the degradation test, the cylindrical shaped hydrogels likewise samples for mechanical test were prepared and after 1 day, the samples were immersed in 10 mL PBS overnight to reach equilibrium swelling state until 24 h. Then, the samples were immersed in 10 mL PBS containing proteases (1 units/mL of collagenase and hyaluronidase) and also absence of these enzymes. The aliquot solution was refreshed every 3 days. Changes in the hydrogel weight were measured to evaluate the biodegradability and stability and in different time lapses. The degree of weight loss was measured by using the following formula where *W*_0_ and *W*_t_ are the weight of the hydrogel before and after immersing in buffer solution respectively.

1 $${\rm{Weight}}\,{\rm{loss}} \% =\frac{{\rm{W}}0-{\rm{Wt}}}{{\rm{W}}0}\times 100$$

### Release of Dex in vitro

The percentage of Dex loading capacity (LC) and encapsulation efficiency (EE) were measured using the following formula according to a previous report [12, 40]. The prepared Dex-HA-Tyr microparticles were lyophilized and then weighed. The specific amount of dried microparticles was treated with hyaluronidase 50 units/mL for 30 min, resulting in the cleavage of glycosidic bonds of the HA structure and the liquefaction of the HA-Tyr microparticles. The amount of encapsulated Dex was determined by UV–visible spectroscopy at 242 nm using calibration curve based on beer-lambert equation for Dex at determined concentrations.

2 $$LC \% =\frac{{\rm{Weight}}\,{\rm{of}}\,{\rm{encapsulated}}\,{\rm{Dex}}\,{\rm{in}}\,{\rm{microparticles}}}{Weight\,of\,the\,microparticle\,containing\,Dex}\times 100$$

3 $$EE \% =\frac{{\rm{Weight}}\,{\rm{of}}\,{\rm{encapsulated}}\,{\rm{Dex}}\,{\rm{in}}\,{\rm{microparticles}}}{{\rm{Weight}}\,{\rm{of}}\,{\rm{the}}\,{\rm{initial}}\,{\rm{Dex}}}\times 100$$

To measure the release rate of Dex from synthesized MPs and composite system, a specific amount of samples was immersed in 50 mL of PBS within a corning tube with constant stirring on arbitrary shaker at 50 rpm. The aliquot of the buffer solution was periodically harvested for analysis at different time intervals and replaced with fresh PBS. The concentration of Dex was determined using UV–visible spectroscopy, and its cumulative release percentage was calculated as the mean ± SD. Additionally, the release kinetics of Dex from the hydrogel samples were analyzed following a previously established methodology [12, 20, 21]. The release profile was validated using the Korsmeyer-Peppas model and linear fitting models, including Zero-order, First-order, Higuchi, and Hixson-Crowell models. The regression coefficient (*R*^2^) values were determined to identify the most appropriate fitted model [12, 20, 21].

4 $${\rm{Korsmeyer}}{\hbox{-}}{\rm{Peppas}}\!:{{\rm{M}}}_{{\rm{t}}}/{{\rm{M}}}_{\infty }={{\rm{K}}}_{{\rm{p}}}{{\rm{t}}}^{{\rm{n}}}$$

5 $${\rm{Zero}}{\hbox{-}}{\rm{order}}\!:{{\rm{Q}}}_{{\rm{t}}}={{\rm{Q}}}_{0}+{{\rm{K}}}_{0}{\rm{t}}$$

6 $${\rm{First}}{\hbox{-}}{\rm{order}}\!:\,\mathrm{ln}\,{{\rm{Q}}}_{{\rm{t}}}=\,\mathrm{Ln}\,{{\rm{Q}}}_{0}+{{\rm{K}}}_{1}{\rm{t}}$$

7 $${\rm{Higuchi}}\!:{\rm{Q}}={{\rm{K}}}_{{\rm{H}}}{{\rm{t}}}^{1/2}$$

8 $${\rm{Hixon}}{\hbox{-}}{\rm{Crowell}}\!:{{\rm{Q}}}_{0}^{1/3}-{{\rm{Q}}}_{0}^{1/3}={{\rm{K}}}_{{\rm{s}}}{\rm{t}}$$

In Korsmeyer-Peppas equation, *M*_t_ is the accumulative amount of drug released at time *t*, *M* ∞ , the initial drug loading, *K*_P_, a constant characteristic of the drug-polymer system, and *n* represents the diffusion exponent which implies nature of the release mechanism. In addition, *Q*_t_ is the quantity of drug dissolved at time *t*, *Q*_0_ is the initial quantity of drug in the solution (most of the time, *Q*_0_ = 0), *K*_0_ is the zero-order release constant, *K*_1_, the first-order release constant, *K*_H,_ the Higuchi dissolution constant, and *K*_s_ is a constant incorporating the surface-volume relation in Hixon-Crowell model [12, 20, 21].

### Proliferation of encapsulated cells

ADSCs 0.5 × 10^6^ cells/mL were added to the 0.5 mL Gela precursor hydrogel solution. Then, the Dex-HA-Tyr Mps and PA solutions were respectively added to the sample and after quick homogenization, were placed on a 24-well cell culture plate for cytocompatibility and proliferation analyses. The cellular morphologies were observed by using an immunofluorescence microscope (Olympus BX51, Japan). In addition, the cellular proliferation was determined to notice the metabolic activity of the encapsulated ADSCs in the designed hydrogels by a convenient WST-1 assay at different time points. For this, 1 mL of WST-1 solution was added to these hydrogels and incubated for 6 h under physiological conditions on a rotary shaker to homogenize the reactant. The supernatant medium was then collected and the amount of water-soluble formazan dye derived from tetrazolium salt dissolved in the medium was measured at 450 nm using a spectrophotometer against blank background control. The collagen gel matrix was used as a consistent control group for the prepared Gela-PA hydrogels.

### Sciatic injury in animal model

**Fig. 1:**
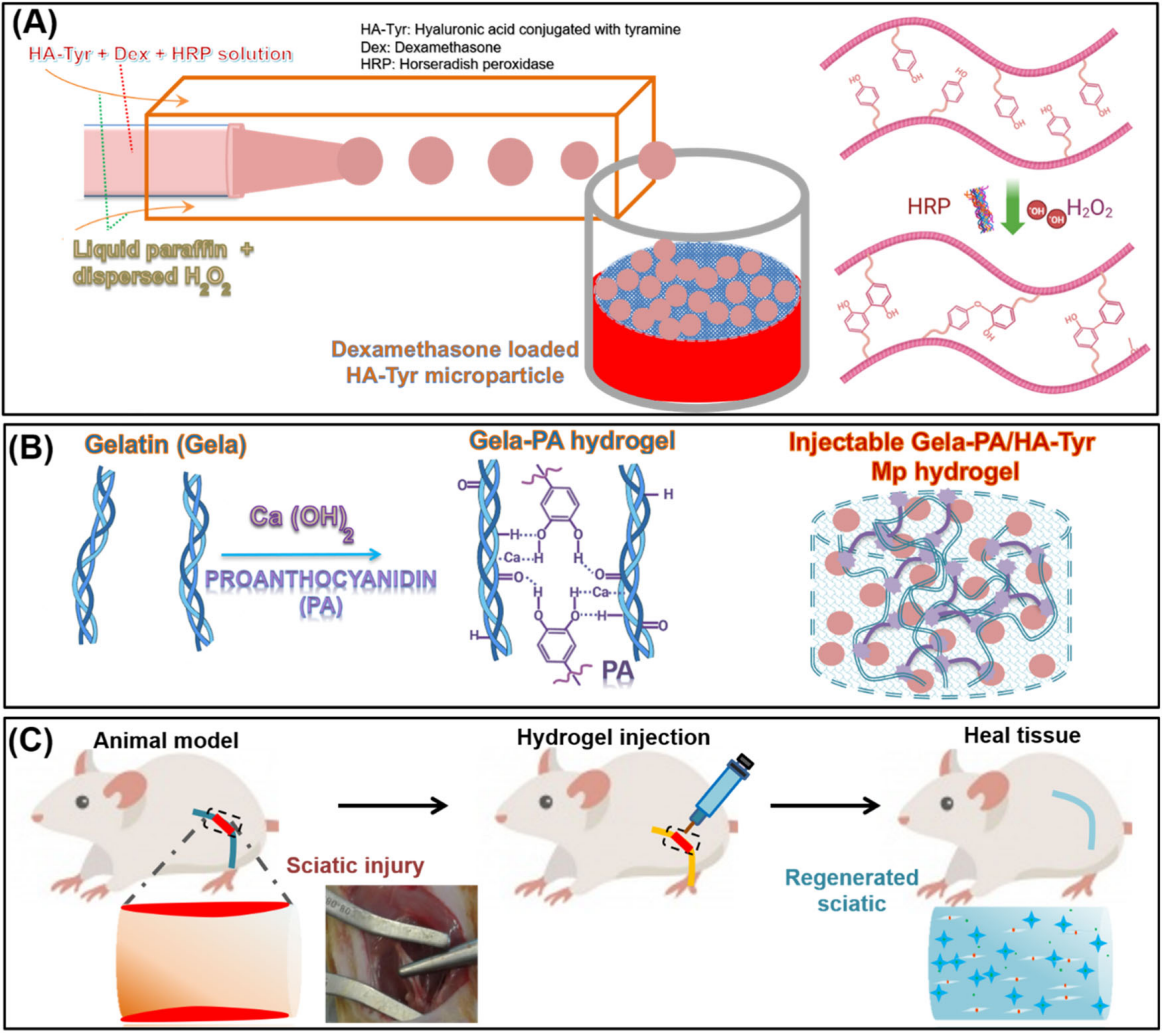
Schematic illustrations of (**A**) preparation of dexametasone loaded HA-Tyr microparticles (Dex-HA-Tyr Mps) though horseradish peroxidase mediated crosslinking using microfluidic device (**B**) gelatin (Gela)-based hydrogel preparation through proanthocianidine (PA). **C** sciatic tissue injury in rat model and treatment with prepared hydrogels for nerve tissue regeneration

Twenty-four healthy Sprague rats (mean body weight of 250 g) were housed and kept at the institutional animal center 2 weeks before study and libitum in individual ventilated cages. The rats were randomly divided into four groups including: (1) injection of Dex-HA-Tyr Mp, (2) injection of Gela-PA, (3) injection of Gela-PA/Dex-HA-Tyr Mp, and (4) negative control (with injury but no surgical intervention). Rats were anesthetized with ketamine and xylazine by intraperitoneal injection of 60 mg Ketamine 5%/10 mg Xylazine 2%/kg body weight. Sciatic nerve tissue crush was performed according to the protocol reported by Beer et al. [41]. For this, a long skin incision was made on the left lower limb of the rats and the subcutaneous skin pocket was dissected through a few mm-long crush injury, 1 cm above the bifurcation of the tibial and peroneal nerves which was made by exerting a constant force using a non-serrated clamp for a period of 30 s (Fig. 1). The prepared hydrogel sample was injected locally at the site of injury using a 22-gauge needle connected to a syringe and in the following, musculature and also skin were sutured with polyglycolic acid and silk sutures respectively.

### Sciatic function index (SFI)

Recovery of motor function was examined using the sciatic function index (SFI) test according to the standard protocol at indicated times of post-surgery [3, 4]. For this, the rats were allowed to walk down on a walking alley (100 × 10 × 10 cm L × D × h) in a darkened chamber with bHRPk and white papers at the end and floor, respectively. Their hind feets were immersed in the ink to mark their positions during walking on the papers and record their footprints. The sciatic functional index (SFI) was measured by using the below equation:

9 $$\begin{array}{l}{\rm{SFI}}=-38.3({\rm{EPL}}-{\rm{NPL}})/{\rm{NPL}}+0.95({\rm{ETS}}-{\rm{NTS}})/{\rm{NTS}}\\ \qquad\quad+\,13.3({\rm{EIT}}-{\rm{NIT}}) /{\rm{NIT}}-8.8\end{array}$$

Here, E and N marks are, respectively experimental and normal feet. The PL and TS are the distance between the heel and the top of the third toe and from the first toe to the fifth toe respectively. Also, IT is the length between the second toe and the fourth toe. The SFI value for totally impairment and normal rats were determined −100 and 0, respectively.

### Hot plate test

Eight weeks after surgery, the recovery of sensory function was performed by the hot plate latency examination [3, 42]. The injured animal limb was positioned on a hot plate at 56 °C and the time until animals responded by licking their paws or jumping was recorded as a response time to heat. The cut-off time for their responses was set at 10 s.

### Histopathologic and immunohistochemical characterization

The injured sciatic nerve tissue was harvested for histopathological investigation after twelve weeks of post-surgery. The animals were sacrificed through carbon dioxide asphyxiation, and injured sciatic tissues were harvested and immersed in 10% (v/v) buffered formalin. Then, the samples were gradually dehydrated and then embedded in paraffin blocks for tissue sectioning. Afterwards, the samples were sliced into 6 µm cross sections using rotary microtome. The prepared film was fixed on glass slide and deparaffinized and then rehydrated before applying staining protocol. The treated slides were stained by hematoxylin and eosin (H&E) as well as luxol fast blue (LFB) by following standard protocol. Furthermore, the parenchyma infiltration of inflammatory cells and perivascular cuffing cells were analyzed from H&E microphotographs. Besides, the demyelination was evaluated by LFB which detects the myelin blue degrees using microscopic observation.

Neurofilament cells are the major components of the neuronal cytoskeleton and play an important role in axon proliferation and outgrowth. Anti-NF-200 antibody staining was used for the immunofluorescence analysis. Tissue sections were stained for NF-200 with an anti-NF antibody (Duccio-Artiuo, Abcam, USA) that reacts with the 200-kDa neurofilament protein. Secondary antibody was added to the samples and in the following these were stained with DAB staining. The samples were counterstained with hematoxylin and analyzed under an inverted light microscope.

### Statistical analysis

The data are shown as means ± standard deviation (SD). Statistical analysis was carried out by Minitab 18 software (Minitab, Inc., State College, USA). Significant differences are expressed as *P value < 0.05, **P value < 0.01 and ^ns^*P* value > 0.05 in bar graphs.

## Results and discussion

### Microparticle characterization

**Fig. 2:**
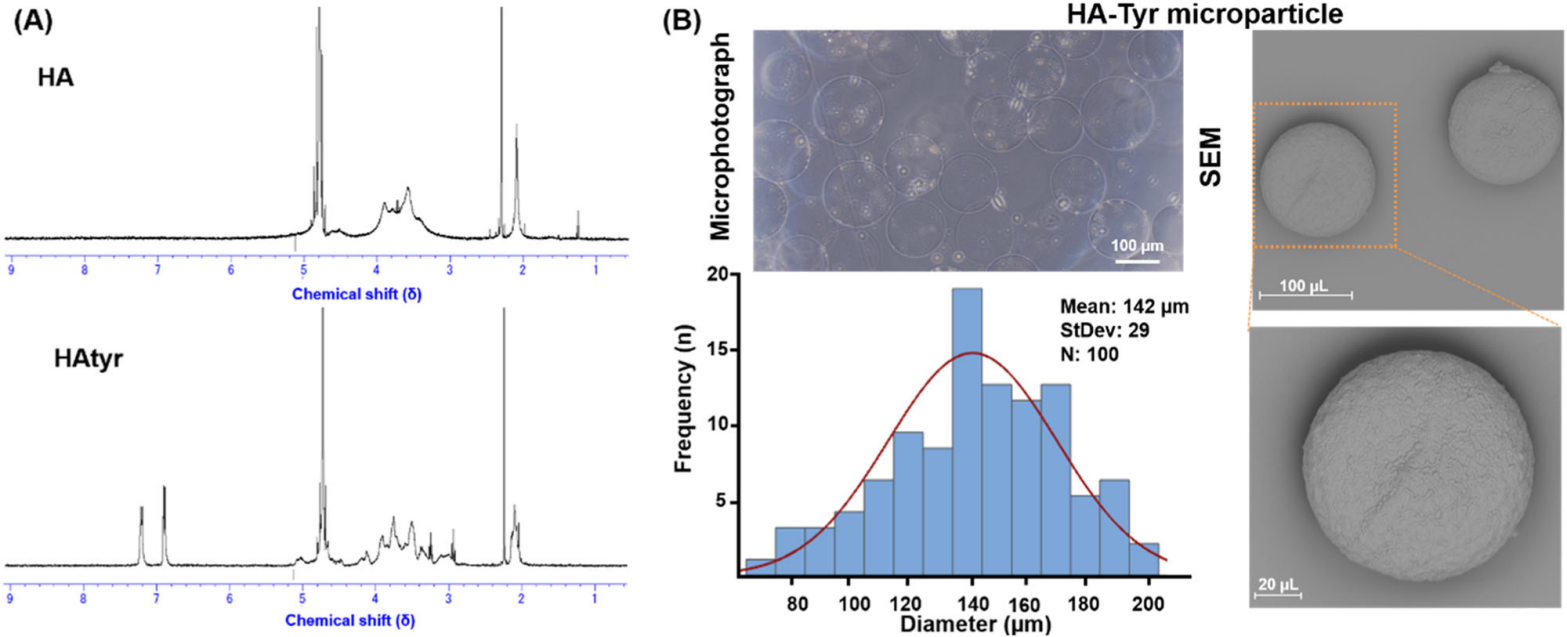
**A** ^1^HNMR of of HA and syhtesized HA-Tyr. **B** Representative microphotograph and SEM of hydrogel microparticle from HA-Tyr and their size distribution

The successful conjugation of Tyr moieties in the backbone of the synthesized HA-Tyr was confirmed by ^1^HNMR and UV-visible spectrophotometry [18, 28, 36]. The distinctive peaks identified at 6.8–7.4 ppm corresponded to protons of Ph moieties at ortho and meta positions (Fig. 2A). Also, the Tyr moiety content in HA-Tyr was 2.1 × 10^−4^ (mol Tyr/g HA-Tyr). We employed a microfluidic-based microparticle fabrication whereby HA-Tyr solution stream co-flowed in a coaxial manner where discretised into droplets by ejection into an oil stream. The focus of our research was to reveal the controlled release of Dex from biocompatible microparticles as a useful substrate in regenerative medicine, particularly for nerve tissue. We prepared Dex-HA-Tyr Mps by HRP-catalyzed crosslinking using a coaxial microfluidic device. Droplet uniformity is a well-known advantage of flow-focused microfluidic system, which makes it a beneficial vehicle for sustained drug/cell delivery without discrepancy concerns among vehicles [8, 21, 28].

Here, within the microfluidic device, when two fluids meet, first an interface is formed due to the immiscibility of the inner and outer fluids. Then, shear force pushes the head of the dispersed fluid into the continuous fluid until stream breaks off and the droplet is generated [8, 14]. Then it circulates in the channel tube of the continuous fluid and suspended in collecting tube. H_2_O_2_ diffused into aqueous droplets, and crosslinking among Ph groups was proceeded by an HRP-mediated reaction [4, 21, 28]. The prepared droplets were uniform and in narrow size distribution. After crosslinking time and washing droplets with PBS, the obtained microparticles showed suitable sphericity and homogeneous structure in narrow size at 142 ± 29 µm (Fig. 2B). Besides, the surface of the prepared microparticles was relatively uniform and smooth, which is the benefit of oil emulsion system and homogeneity of crosslinking among HA-Tyr molecules for prepared polymeric droplets (Fig. 2B).

### FTIR investigation

#### FTIR of Dex and HA-Tyr microparticle

**Fig. 3:**
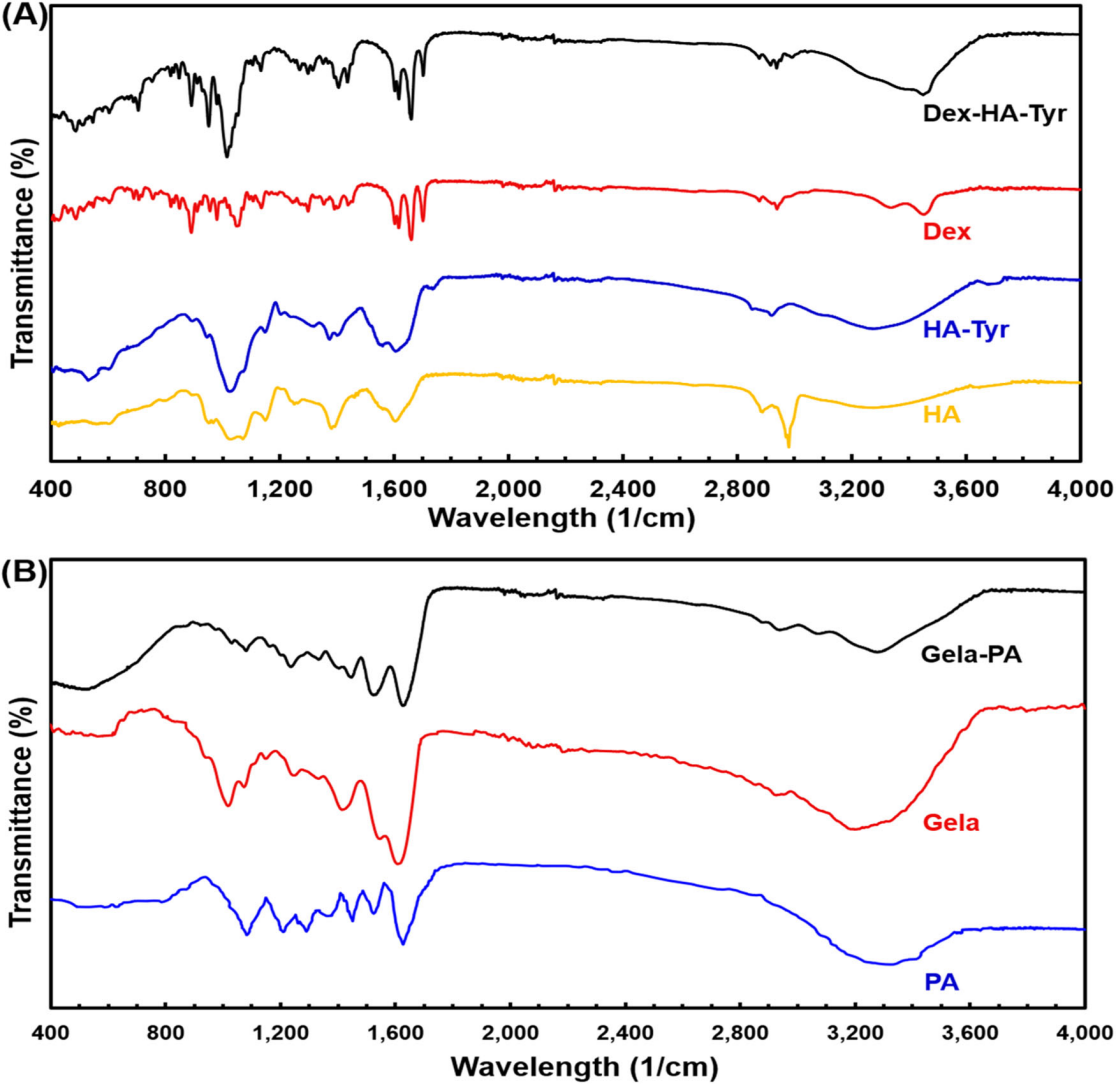
FTIR spectra of (**A**) HA, HA-Tyr, Dex, and Dex-HA-Tyr microparticle, (**B**) PA, Gela, and Gela-PA hydrogel

In Dex spectrum, the peak at 1055 cm^−1^ corresponds to the P-O-C band, and it is the main characteristic of dexamethasone [20, 43]. Moreover, the peaks at 1705 cm^−1^ and 1665 cm^−1^ are attributed to carbonyl (C=O) absorption band and -COO stretching band, respectively (Fig. 3A). HA shows peaks of C-O-C stretching and C-O group with C–O combination (COO ester) at 608 cm^−1^ and 1072 cm^−1^, respectively (Fig. 3A). Moreover, the peak at the 1656 cm^−1^ belongs to amid carbonyl (amide II) group. The NH and OH stretching can be observed at 3337 cm^−1^. The peaks at 2979–2841 and 1395 cm^−1^ are assigned to C-H stretching vibration and bending, respectively. The peak at 1418 cm^−1^ belongs to the stretching of COO− which refers to the HAs acid groups. Finally, the stretching region of group protonated COOH is also observed at 1258 cm^−1^ (Fig. 3A). In addition, HA-Tyr shows a broader peak with lower intensity in the region of 3500–3000 cm^−1^ as an indication of the interaction between HA and Ph. There are also shifts in the amine groups of the HATyr to 1622 cm^−1^ due to the presence of the Tyr amine group and the appearance of weak new absorption peaks at 534 and 3708 cm^−1^ which shows the presence of phenol groups in the structure (Fig. 3A) [43, 44]. The incremental change intensity observed at 1661, 1440, 1401, 1123, 1019, and 545 cm^−1^ can also be related to the combinational effect of the Dex and HA-Tyr components, confirming the presence of both of them in the microparticle structure [20, 43, 44]. By the analysis of FTIR spectra (Fig. 3A), one can observe that the characteristic absorption bands related to dexamethasone could be found in the spectra of impregnated matrices when compared to the spectrum of dexamethasone alone (Fig. 3A).

#### FTIR of PA and Gela-PA hydrogel

The absorption peaks at 3264 and 2947 cm^−1^ are assigned to amide A and amide B, respectively. These peaks are mainly associated with the stretching vibrations of N–H groups (Fig. 3B). The peaks at 1610, 1571, and 1251 cm^−1^ are attributed to the distinctive vibrations of amide I (stretching vibrations of peptide C=O groups), amide II (N–H bending vibrations coupled to C–N stretching vibrations), and amide III bands (C–N stretching and N–H bending vibrations, as well as wagging vibrations of CH_2_ groups in the glycine backbone and proline side chains), respectively (Fig. 3B) [8, 13, 34]. The increase in procyanidin content did not result in a significant change in the positions of these major amide bands. However, only the amide A, I, and II bands were partially broadened, which involves hydrogen bond interactions between procyanidin and Gela molecules [13, 34]. The minor alteration in the chemical shift of the amide I band, shifting from 1610 to 1633 cm^−1^ after adsorption of proanthocyanin onto the Gela-based hydrogel, indicates hydrogen bonding interaction (Fig. 3B). These changes indicating the incorporation of PA and thus the generation of PA crosslinking is in agreement with related reports [31, 33, 34]. It is widely accepted that hydrogen bonding plays a dominant role in stabilizing protein-based scaffolds by plant polyphenols under acidic conditions. The relatively minor differences in the dipole character of individual phenolic substances seem to influence their binding to this biopolymer. Indeed, the side chain hydroxyl, carboxyl, amino, and amide groups of gelatin molecules provide potential interaction sites for hydrogen bond formation. Indeed, the side chain hydroxyl, carboxyl, amino and amide groups of gelatin molecules provide the potential interacting sites for the formation of hydrogen bonds [31, 33, 34, 45]. These typical band complexes of amide were also obviously observed in the Gela-based hydrogel, with peaks at 1633 (1610), 1535 (1571), and 1244 (1251) cm^−1^ for their amide I, II, and III bands, respectively. The amide A and B bands at 3302 cm^−1^ and 3091 cm^−1^, respectively, are mainly associated with the stretching vibrations of N–H groups (Fig. 3B).

### Mechanical strength and stability

**Fig. 4:**
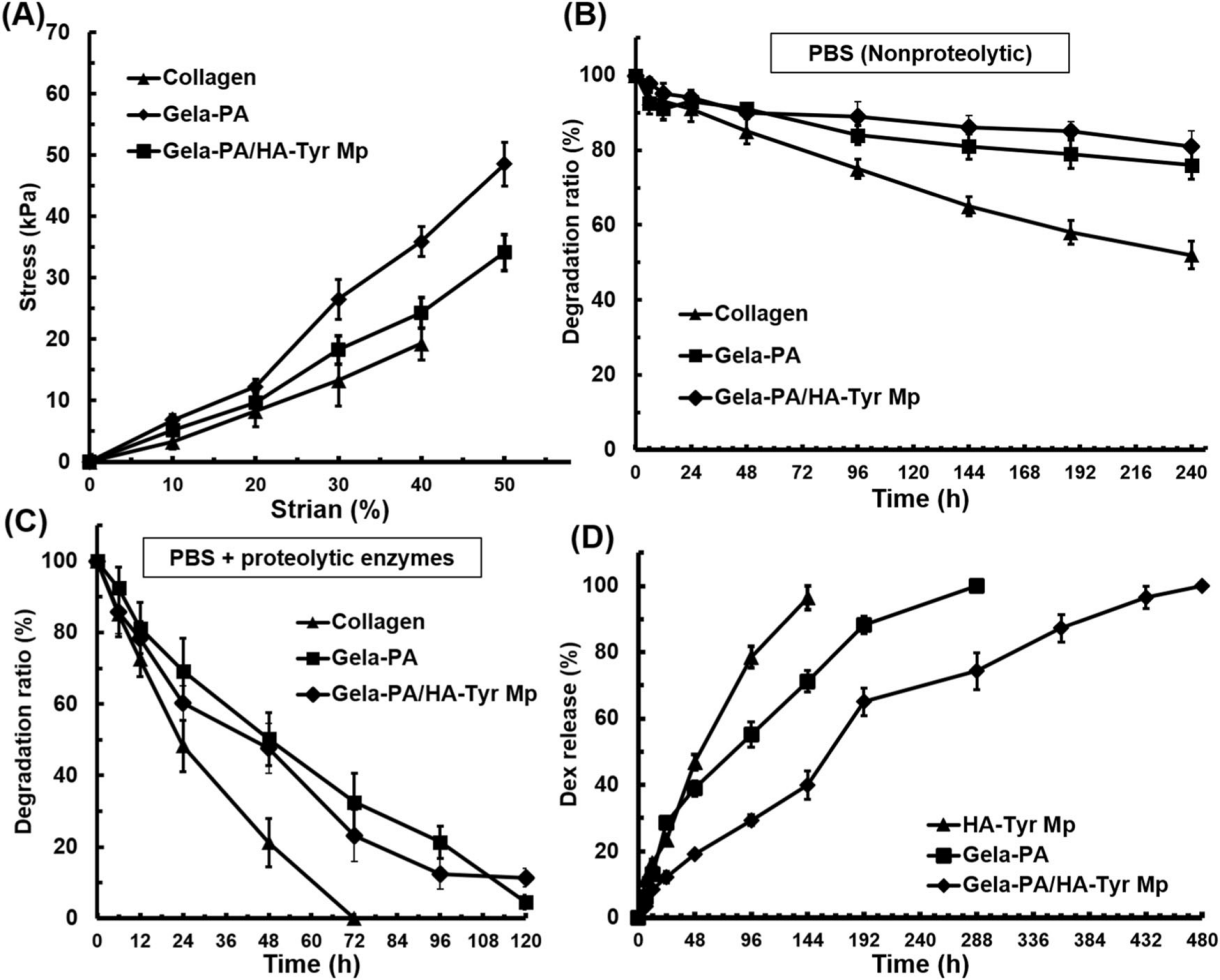
Physical properties of hydrogels. **A** Load-compression profile of hydrogels. **B**, **C** Weight loss rate of the hydrogels in PBS in the presence and absence of proteolytic enzymes, respectively. **D** Controlled release of Dex form hydrogel constructs in different conditions, including HA-Tyr Mp, Gela-PA, and Gela-PA/HA-Tyr Mp. Vertical bars: SDs (*n* = 4)

As shown in Fig. 4A, the hydrogel constructs, including Gela-PA, Gela-PA/HA-Try Mp, and collagen (control), were tested under compressive stress-strain profiles up to 50% strain. Unlike collagen gel whose maximum strength was 18 kpa and ruptured at 42% stain, the Gela-PA hydrogels were proven to be resistant to pressure and showed 48 kPa strength. Meanwhile, the incorporation of HA-Tyr Mp within Gela-PA hydrogel resulted in lower mechanical stability and hydrogel displayed 32 kPa strength at 50% strain.

In addition, the stability of hydrogel composites was evaluated by soaking cylindrical hydrogels in saline buffer with and without proteolytic enzymes. It was observed that the degradation of the hydrogels progressed at an accelerated rate as the incubation period extended in longer time. Meanwhile, degradation index for collagen hydrogel reached to 48% over 240 h incubation in PBS without proteolytics **(**Fig. 4B, C). The degradation index for Gela-PA hydrogels did not exceed 23%, highlighting the influence PA-calcium hydroxide crosslinker on mitigating hydrogel degradation (Fig. 4B). Interestingly the hydrogels completely degraded in presence of proteolytic media within 120 h (Fig. 4C). Furthermore, it is worth noting that the rate of weight loss in Gela-PA hydrogels was consistently lower than that of collagen gel across all time points. Nevertheless, both hydrogels exhibited complete degradation within 120 h. This observation underscores the effectiveness of PA-calcium hydroxide as a cross-linking agent in enhancing the stability of gelatin-based hydrogels.

### Encapsulation efficacy and release profile of Dex

The drug delivery system with a suitable release profile needs to be carefully selected to achieve a favorable pharmacological treatment [20–22]. The LC% and EE% of encapsulated Dex for prepared HA-Tyr Mp were quantified by proteolytic degradation of HA-Tyr molecules using hyaluronidase. The LC% and EE% were determined 5.3 ± 0.3% and 81.2 ± 4.9%, respectively which exhibited successful encapsulation of Dex in HA-Tyr Mp. It is expected that a particular amount of used Dex in the Mp fabrication process would be extracted from the microparticles through washing steps to remove oil from the surrounding of MPs, and this would cause a relative reduction of the EE% index [20, 43]. Morevoer, the cumulative release profile of encapsulated Dex showed the release of 19.1 ± 3.4% and 29.4 ± 2.7% in the first 12 and 24 h, respectively, followed by a moderate release profile up to 97.4 ± 2.1% over 6 days, and after that there was no significant difference between time-courses, proving that all of the Dex molecules diffused out and released from HA-Tyr Mp (Fig. 4D). The Gela-PA hydrogel displayed slower release profile and all of the encapsulated Dex molecules became free in the supernatant media within 14 days of incubation (Fig. 4D). The designed hydrogel structure prolonged the release profile of Dex for more than 18 days which is significantly higher than single HA-Tyr Mp as well as Gela-PA hydrogel networks (Fig. 4D).

To investigate the release mechanism of Dex in the Mp and hybrid of Mp and Gela-PA hydrogel, the accumulative release profiles were examined by the Korsmeyer-Peppas equation and the zero-order, first-order, Higuchi, and Hixon-Crowell models and for these, the linear fitting results as well as the regression coefficients (*R*^2^) are reported in Table 1. As is observed, according to the Korsmeyer-Peppas model, the drug transport mechanism of both HA-Tyr Mp and Gela-PA conditions displayed fickian diffusion, and Gela-PA/HA-Tyr Mp demostrated anomalous transport. As the release of pharmaceutical polymeric dosages with limited recognition is only valid for “n” values less than 0.6. It is important to highlight that Gela-PA and partially HA-Tyr Mp stands out with a notably rational regression coefficient in this context. The high regression coefficients for the samples indicate fit with the zero-order model, suggesting a release of the low soluble drug into the release environment. Also, due to Dex adsorption in PBS, all the samples exhibited a strong fit with the Higuchi model, characterized by high regression coefficients. This result suggests that drug diffusion outpaces matrix degradation and leads of drug release primarily via a diffusion mechanism rather than being constrained by matrix degradation [20, 21, 43]. Besides, the first-order and Hixon-Crowell models exhibit a low coefficient which suggests a limited drug release from the samples due to the degradation of substrates [20–22, 43].

**Table 1:** The regression coefficient of kinetic models fitted for release of encapsulated Dex from HA Mp and Gela-PA hydrogel

Samples	Korsmeyer-Peppas			Zero-order		First-order		Higuchi		Hixon-Crowell	
	*K*_p_ (h^−n^)	*n*	*R*^2^ (-)	*K*_0_ (μg.mL^−1^.h^−1^)	*R*^2^ (-)	*K*_1_ (h^−1^)	*R*^2^ (-)	*K*_H_ (μg.mL^−1^·h^−1/2^)	*R*^2^ (-)	*K*s (μg ^1/3^.mL^−1/3^.h^−1^)	*R*^2^ (-)
HA-Tyr Mp	0.036	0.61	0.96	8.2	0.97	0.015	0.76	55.6	0.92	−1.99	0.86
Gela-PA	0.033	0.57	0.94	7.1	0.93	0.011	0.79	45.3	0.94	−1.69	0.73
Gela-PA/HA-Tyr Mp	0.005	0.79	0.92	2.7	0.92	0.002	0.68	24.1	0.91	−1.4	0.76

### Cell behavior in hydrogels

**Fig. 5:**
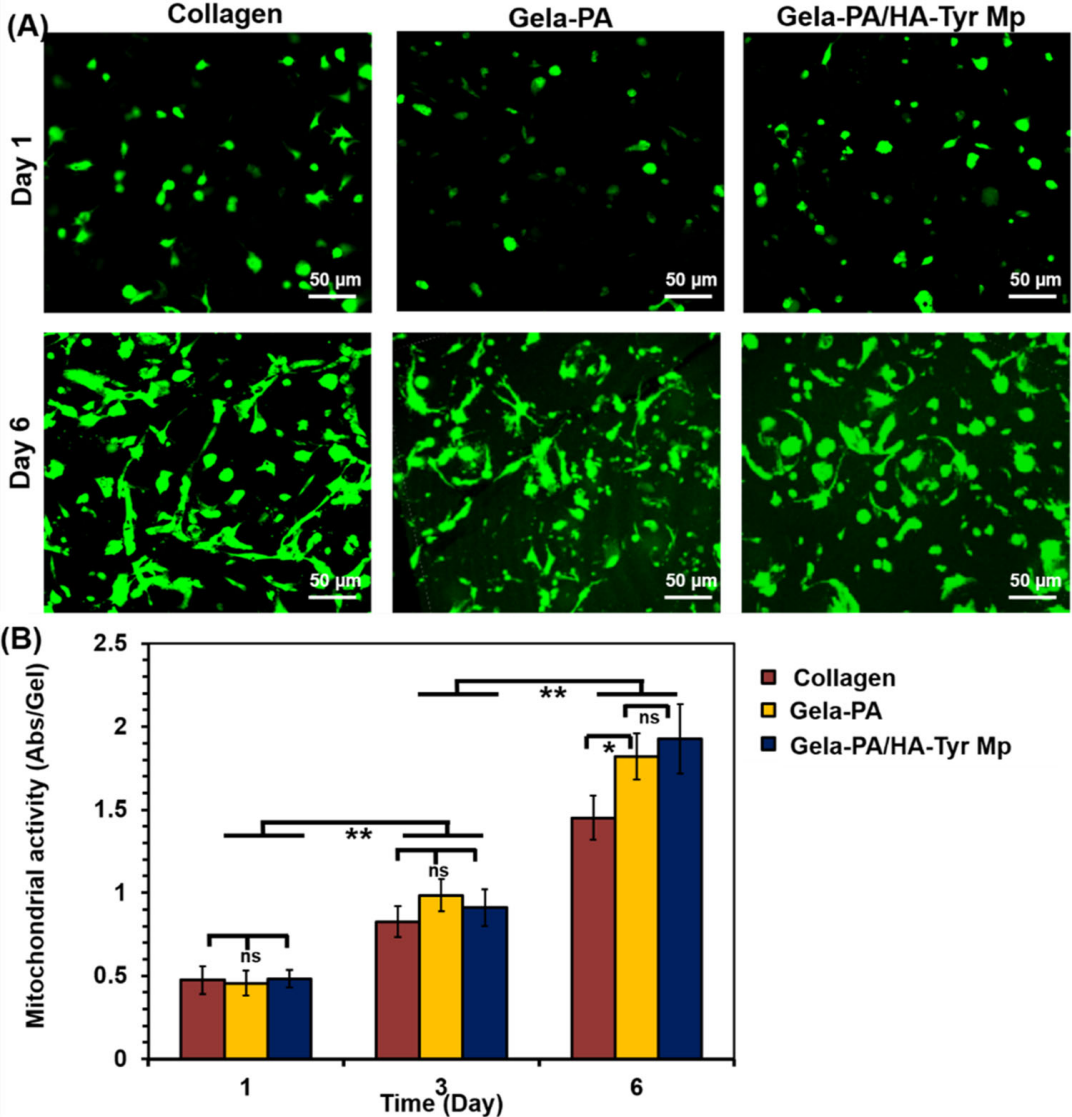
Morphology (**A**) and proliferation (**B**) of encapsulated adiposed derived stem cells in different hydrogel composites at the indicated time points. Error bars: standard deviations (SDs, *n* = 3–4)

Cellular viability was measured by trypan blue exclusion assay through proteolytic degradation of gels by using collagense. The results showed that encapsulated cells were significantly viable at 91.2 ± 6.1% for Gela-PA, 93.5 ± 4.5% for Gela-PA/HA-Tyr Mp and 89.9 ± 4.4% for collagen hydrogels which suggested that encapsulation process did not insert specific deleterious impacts on cell survival and viability [31, 32]. Morphologies of encapsulated ADSCs in the developed hydrogels monitored and characterized using a fluorescence microscope. As can be seen in Fig. 5A, the density of encapsulated cells increased during extension of culture time. We can observe a stretched morphology for cells with very good dispersion in both Gela-PA hydrogel conditions with and without HA-Tyr Mps, likewise the collagen gel group (Fig. 5A).

The growth ability of the cells was measured by the mitochondrial activity analysis. Figure 5B shows the mitochondrial activity of ADSCs within the hydrogels at different time points. It can be seen that the Gela-PA hydrogels with and without HA-Tyr Mps had the same level of mitochondrial activities at day 1 after encapsulation. However, the mitochondrial activity at day 6 shows the highest value for the Gela-PA sample compared to the collagen gel as a control condition.

### Sciatic function index (SFI)

**Fig. 6:**
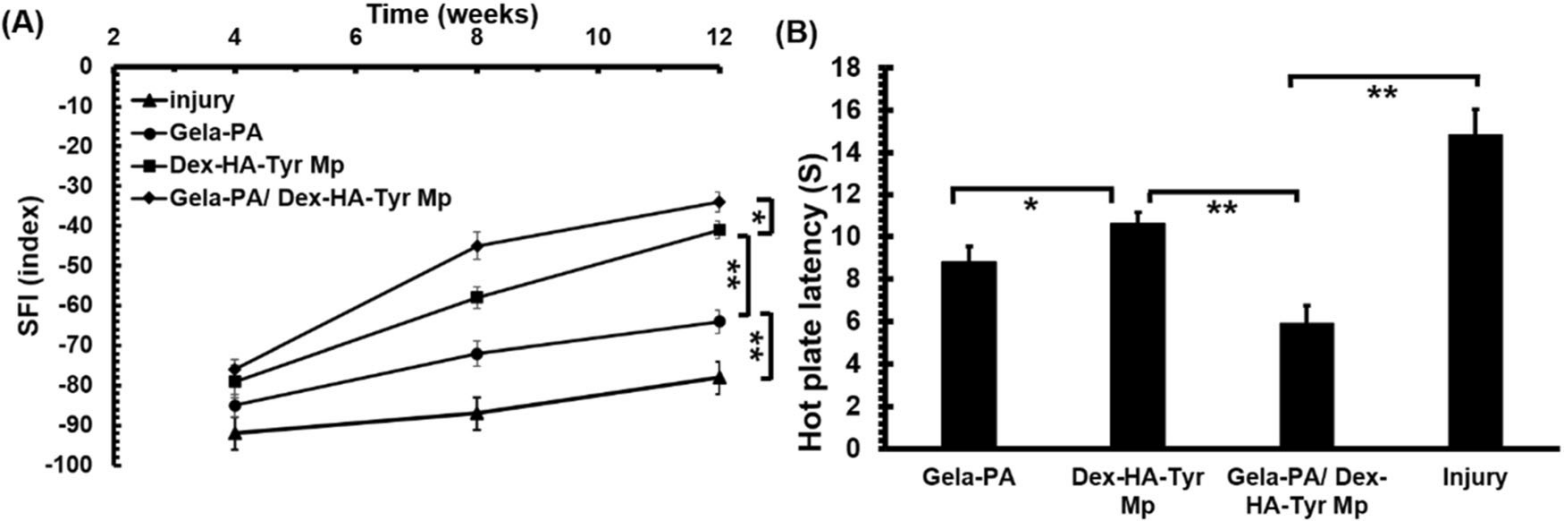
**A** Sciatic functional index study results of different groups 4, 8, and 12 weeks post-surgery. **B** Hot plate latency time measurement results of different groups respectively 12 weeks post-surgery

The average sciatic functional index (SFI) values of all study groups. The negative control had an average SFI value −91.4 ± 3.2 after 4 weeks and this index did not change significantly during the extended time of study and reached to 81.1 ± 3.9 at the end of the 12th week (Fig. 6A). The Dex-HA-Tyr Mp and Gela-PA hydrogel treatment conditions considerably enhanced the SFI value from −79.4 ± 2.9 and −85.1 ± 3.1 at the end of week 4 to −40.1 ± 3.1 and −64.2 ± 2.7 at the end of week 12, respectively. The SFI values for the Gela-PA/Dex HA-Tyr Mp were significantly higher than the other groups at the end of each time period up to the 12-week analysis. The SFI value for Gela-PA/Dex HA-Tyr Mp was −34.2 ± 2.6, showing a more than 105% higher regeneration index compared to the negative control condition (Fig. 6A).

### Hot plate latency test

Evaluation of the nociceptive function of the injured limb was performed by the hot plate latency analysis (Fig. 6B). Rats in the negative control group did not withdraw their paw from the hot plate within 14.5 s. The latency for the Dex-HA-Tyr Mp and Gela-PA/Dex HA-Tyr Mp was recorded a 9.6 ± 0.4 s and 5.4 ± 0.6 s, respectively, which was significantly shorter than the latency for Gela-PA hydrogel (10.6 ± 0.4 s) and the negative control group.

### Histological analysis

**Fig. 7:**
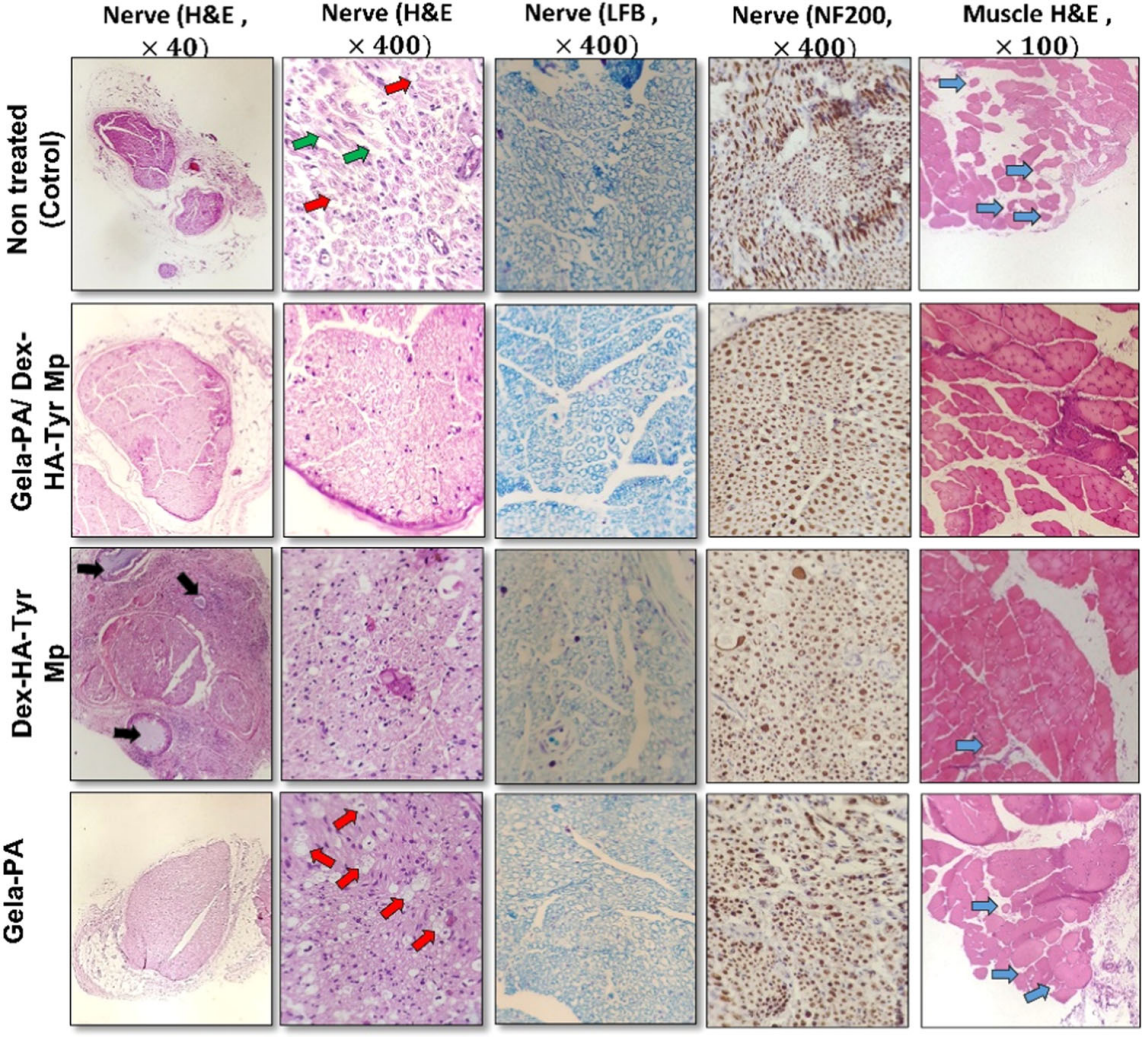
Light micrographs of the sciatic nerve and gastrocnemius muscle in the experimental groups, black arrows: hydrogel, red arrows: vacuolation, green arrows: myelin destruction, blue arrows:n atrophied muscle fiber, H&E, LFB, and NF2-IHC staining

The non-treated group (control) had impaired fiber arrangement with swollen axons as well as varying degrees of vacuolation (Fig. 7). In addition, various severe injuries such as degenerated nerve fibers, significant edema of nerve fibers, myelin sheath disintegration, and axonopathy were observed in this group. These observations indicated that the resulting injury could not be repaired without any treatment. In the Gela-PA/Dex-HA-Tyr Mp group, the nerve fibers were symmetrically distributed and the thickness of the myelin sheath appeared normal (Fig. 7). The biocompatibility of this hydrogel was acceptable and this finding was confirmed in all treated rats with this Gela-PA/Dex-HA-Tyr Mp hydrogel.

The histopathological examination of the Dex-HA-Tyr Mp group revealed a substantial enhancement in the regeneration of myelin sheaths and the overall condition of nerve fibers. Notably, any signs of nerve damage were entirely eliminated, leaving only minimal evidence of vacuolation. However, remnants of the Dex-HA-Tyr Mps were still discernible in this group, and the nerve fibers were encircled by fibrotic tissue. In the Gela-PA treatment group, the histopathological analysis displayed a more pronounced and severe degeneration, marked by significant vacuolation and damage to the myelin sheaths (Fig. 7). The histopathological evaluation of the Gela-PA treated sciatic nerves was partially similar to the Dex-HA-Tyr Mp condition. However, in this group, the irregularity and atrophy of the muscle fibers and the fibrotic tissue between the muscle cells were higher than in the Dex-HA-Tyr Mp group, which prove the importance of sustained delivery of Dex and hyaluronic acid hydrogel as a drug carrier. In the Dex-HA-Tyr Mp treatment, minimal atrophied muscle fibers and fibrosis were observed. Interestingly, in the Gela-PA/Dex-HA-Tyr Mp hydrogel group, collagen fibers were observed between muscle fibers and the muscle fibers looks normal (Fig. 7).

## Discussion

The necessary requirements for developing the next generation of biomaterials in sciatic nerve regeneration applications will be met by engineering a fully degradable construct with excellent mechanical and biological properties [46–48]. Until now, a wide range of natural-based hydrogels, such as gelatin, hyaluronic acid, alginate, fibrin, and collagen, have been studied for sciatic tissue regeneration and repair. However, their applications have not been fully approved due to their weak mechanical properties and fast degradation rate [11, 48]. Therefore, optimizing hydrogel characteristics to obtain bioactive scaffold with physical and biochemical properties similar to the target tissue is one of the important factors in efficient tissue regeneration [10, 11, 48]. The PA crosslinker can improve stability and biological properties of hydrogels [31, 33, 34, 45]. Here, we aimed to investigate the effects of both Dex-HA-Tyr Mp and PA-mediated gelatin hydrogel as bioactive substrates in peripheral nerve tissue regeneration. Results indicated the successful synthesis of HA-Tyr and corresponding Mp fabrication with relatively smooth surface and uniform size distribution through microfluidic technology and oil emulsion system (Figs. 2 and 3), which can be used as a cargo for encapsulation of drugs and bioactive molecules [4, 25, 49].

Meanwhile, the Gela-PA/Dex-HA-Tyr MP group exhibited lower mechanical stability compared to the Gela-PA group (Fig. 4A). This difference might be attributed to the partial disruption and inhomogeneity of the MPs within the Gela-PA hydrogel matrix. This could have occurred because the Gela-PA solution was not sufficiently viscous, and prior to the completion of hydrogelation, the loaded MPs tended to precipitate. Interestingly, both of these experimental conditions resulted in better mechanical durability and longer degradability compared to the conventional collagen gel condition. These results demonstrate that PA-mediated crosslinking promotes intramolecular bond formation among Gela molecules (Figs. 3B and 4A–C) [32, 34, 45].

The enhanced mechanical property and improved resistance to degradation provided by PA-calcium hydroxide can contribute to the long-term stability and functionality of injectable drug-loaded hydrogels for sustained delivery of bioactive molecules in regenerating tissue. Additionally, the mechanical properties of the prepared injectable Gela-PA based hydrogel were highly comparable to those of injectable hydrogels made from gelatin/agar, silk/gelatin, and fibrin hydrogels used for nerve tissue protection and regeneration. In brief, the mechanical properties of Gela-PA hydrogel were similar to various multi-component hydrogels, collagen-based hydrogels, and even rat sciatic nerves [46–48].

The quantity of Dex in LC% and EE% demonstrates high amount of encapsulation capacity of the utilized water in oil emulsion system in a microfluidic device and efficacy of process in Mp production by HRP-mediated hydrogelation which is fast and proceeds within seconds [24, 26, 35, 39]. It is expected that sequential centrifugation and washing process of Mps for oil removal step could cause fractional elimination of Dex moelcules from HA-Tyr Mps [20, 21, 43].

The incorporation of drug-loaded droplets into injectable hydrogels has attracted considerabble attention in tissue regeneration studies [9, 20, 43]. The release profiles of the experimental groups showed a quite fast release of Dex from Mps compared to other groups which could be due to the higher surface area of Mps and the short distance for diffusion of Dex from Mp matrix to surrounding media [9, 20, 21, 43]. Interestingly, the Gela-PA/HA-Tyr Mp exhibited a moderate bi-phasic profile for Dex release, while phase I was obtained as a burst release state of the Dex present on the Mp surface and is characterized by the diffusion of the Dex from water-filled pores of the polymer matrix. The second phase of the process involves the erosion of the Gela-PA hydrogel matrix [9, 16, 20, 30]. Thus, the obtained results validated the hypothesis of prolonged sustain release profile and delay on burst release of Dex from multi-compartment structures of HA-Tyr Mp and Gela-PA hydrogel. Furthermore, the mathematical model for drug release proved strong fitting of release data with the zero-order model, signifying the release of a less soluble drug into the environment (Table 1). Also, Dex adsorption in PBS leads to robust alignment with the higuchi model, indicating that drug diffusion is the primary release mechanism rather that matrix degradation. The low coefficients in the first-order and Hixon-Crowell models suggest limited drug release due to substrate degradation (Table 1). Therfore, all three hydrogel conditions well fitted in Korsmeyer-Peppas, Higuchi, and zero-order models [20–22, 43].

An important factor that confirmed the success of the proposed fabrication strategy for biocompatible hydrogel composites in tissue engineering and drug delivery applications is the ability of cells to survive and grow [9, 50, 51]. The encapsulated cells in Gela-PA hydrogel spread and proliferated well similar to collagen gel (Fig. 5A) which is attributed to the existence of cell adhesion motifs on the structure of these biopolymers [4, 8, 34, 51]. However, the mitochondrial activity measurements of the samples displayed the highest value for the Gela-PA sample (Fig. 5B). This can be attributed to the partially negative impact of HA-Tyr MPs, which inherently possess non-cell adhesive properties and could hinder cell adhesion and growth. Nevertheless, such HA derivatives would disassociate into small fragments at the injured tissue site, subsequently promoting cellular migration and infiltration, which are desirable phenomena in tissue regeneration. Hence, we can conclude that the use of the prepared Gela-PA hydrogel and the incorporation of Dex-HA-Tyr MPs with it did not have negative impacts on the cellular morphologies and growth capabilities of the encapsulated cells. Therefore, they can be safely used for the repair of sciatic nerve defects [3, 11, 42, 47].

After the administration of Gela-PA hydrogels containing Dex-HA-Tyr MPs, the healing efficacy of the injected hydrogels in rats was investigated through sensory/sensorimotor recovery, motor nerve conduction velocity tests, and histological examination. Walking footprint analysis, which measures SFI, and the hot plate latency test are the most common postoperative experiments to evaluate motor function and sensory recovery, respectively, in sciatic nerve regeneration [1, 11, 42, 52]. The walking track analysis in a rat sciatic nerve lesion model showed significant recovery achieved for the groups treated with hydrogels, especially for the Gela-PA/Dex HA-Tyr MP group, and then for the Gela-PA group (Fig. 6A). This suggests that the injected Gela-PA is involved in sensory regrowth of axons and can significantly improve motor function recovery in the injured sciatic nerve. Additionally, the improved recovery of heat response in the treated groups with Dex-HA-Tyr MPs and Gela-PA/Dex HA-Tyr MPs could be attributed to the promotion of sensory axon regeneration in the regenerated tissue site [1, 11, 42, 52].

This suggests that the injected Gela-PA is involved in sensory regrowth of axons and can significantly improve motor function recovery in the injured sciatic nerve. Additionally, the improved recovery of heat response in the treated groups with Dex-HA-Tyr MPs and Gela-PA/Dex HA-Tyr MPs could be attributed to the promotion of sensory axon regeneration in the regenerated tissue site.

Histopathological analyses of sciatic nerves strongly supported the beneficial impacts of controlled release of Dex from HA-Tyr Mps and specifically in the Gela-PA/Dex-HA-Tyr group compared to the control groups on axonal regeneration due to lesser degraded myelin sheets and reduction of edema extent (Fig. 7). These findings provide reliable insights into the neuroprotective effects of Dex in a sustained delivery state from the developed multi-compartment hydrogel and support the application of these agents in the preclinical treatment of peripheral nerve injury [53, 54]. Additionally, as injectable hydrogels containing drug-loaded MPs are more convenient for practical operations and can flexibly match any specific size and shape of a damaged cavity, the impact of the prepared injectable hydrogel composite can be extended to the regeneration of irregular tissue defects and addressing challenges in hard-to-reach tissue areas.

## Conclusion

In this study, we successfully prepared and evaluated injectable Dex-loaded HA-Tyr Mp and Gela-PA hydrogel for the treatment of sciatic nerve crush injury in rats. These results demonstrate the usefulness of monodisperse microparticles generated by a coaxial microfluidic system and HRP-mediated reaction. The superior biophysical properties of the prepared Dex-loaded microparticles and the surrounding matrix from ECM-derived substrates, along with the long-release profile, ensure the stability of the prepared scaffold and sustained drug release. The prepared Dex-loaded HA-Tyr Mp and Gela-PA hydrogel showed excellent biocompatibility because of the low cytotoxicity of both of HRP and PA-mediated reactions. Animal study results proved that the simultaneous use of both Gela-PA and Dex-loaded HA-Tyr Mp has great potential for sciatic nerve regeneration. From these results, it can be concluded that the generated Dex-HA-Tyr Mp and its surrounding Gela-PA hydrogel are a feasible platform for neural tissue engineering and regenerative medicine.

## Data Availability

Research data are not shared and are available after publication. Please contact the author for data requests.
