# Author Correction: Dexamethasone release from hyaluronic acid microparticle and proanthocyanidin-gelatin hydrogel in sciatic tissue regeneration

**DOI:** 10.1007/s10856-024-06787-x

**Published:** 2024-05-06

**Authors:** Kazem Javanmardi, Hamideh Shahbazi, Ava Soltani Hekmat, Mehdi Khanmohammadi, Arash Goodarzi

**Affiliations:** 1 Department of Physiology, Fasa University of Medical Sciences, Fasa, Iran; 2 Skull-Based Research Center, Five Senses Health Research Institute, School of Medicine, Iran University of Medical Sciences, Tehran, Iran; 3 Faculty of Materials Science and Engineering, Warsaw University of Technology, Warsaw, Poland; 4 Department of Tissue Engineering, School of Medicine, Fasa University of Medical Sciences, Fasa, Iran

Correction to: Journal of Materials Science: Materials in Medicine

10.1007/s10856-023-06768-6 published online 11 January 2024

The original version of this article unfortunately contained a mistake. The author name “Mehdi Khanmohammadi” was incorrectly written as “Mehdi Khanmohamadi”. The original article has been corrected.

